# Dysregulation of the Cant1/β-Catenin/TCF4–CHSY1 Axis Underpins Impaired ECM Biosynthesis in Skeletal Disorders

**DOI:** 10.34133/research.1227

**Published:** 2026-04-01

**Authors:** Yuanliang Li, Wenqi Yu, Yingxin Li, Kai Liu, Wenjing Xu, Cong Li, Yugu Li, Ying Li, Zhaoxin Tang, Yung-Fu Chang, Aoyun Li, Hui Zhang

**Affiliations:** ^1^ College of Veterinary Medicine, South China Agricultural University, Guangzhou 510642, China.; ^2^ College of Veterinary Medicine, Cornell University, Ithaca, NY, USA.; ^3^ College of Veterinary Medicine, Henan Agricultural University, Zhengzhou 450046, China.; ^4^ College of Animal Science, Xizang Agricultural and Animal Husbandry University, Linzhi 860000, China.

## Abstract

Mutations in a specific protein called calcium-activated nucleotidase 1 (Cant1) cause skeletal deformities, but the role of Cant1 in these deformities remains unclear. This study shows how Cant1 acts as a key regulator of bone and cartilage health. We found that Cant1 binds to and stabilizes a protein called Wnt/β-Catenin. Wnt/β-Catenin then enters the cell nucleus to activate specific genes. One of these genes, CHSY1, is turned on to produce building blocks such as collagen and sugars that form the extracellular matrix (ECM), which acts as the scaffolding of cartilage. When Cant1 and Wnt/β-Catenin expression are suppressed, there is a reduction in glycosaminoglycans (GAGs; mucopolysaccharides) and proteoglycans (like ACAN), which create a hydrated, gel-like matrix by binding with hyaluronan and link proteins to make cartilage resistant to compression. Additionally, there is a decrease in the α-1 chain of type II collagen (COL2α1), which forms the structural mesh or framework that gives tissue its tensile strength. In summary, we identified a conserved signaling pathway, the Cant1/β-Catenin/transcription factor 4 (TCF4)–CHSY1 axis, that regulates ECM homeostasis during skeletal development. Dysfunction of this pathway is a core cause of skeletal disorders. These findings not only provide mechanistic insights into human Cant1-related skeletal diseases but also highlight potential new targets for broad-spectrum therapies aimed at correcting deficiencies in ECM biosynthesis.

## Introduction

 Articular cartilage is a highly organized structure that surrounds and protects the ends of bones. It mainly consists of type II collagen, hyaluronic acid, proteoglycans, and chondrocytes. Endochondral ossification, a crucial biological process in cartilage development, plays a key role in bone formation, supporting skeletal growth and maturation [1]. In-depth research shows that important signaling pathways, including Wnt/β-Catenin, Hippo-YAP, transforming growth factor-β (TGF-β), fibroblast growth factor, and bone morphogenetic protein (BMP), important influence chondrocyte differentiation and extracellular matrix (ECM) production [2–4]. Because of the vital functions of chondrocytes in skeletogenesis, abnormal ECM production—particularly in glycosaminoglycan (GAG) biosynthesis—and chondrocyte differentiation are closely associated with the development of metabolic bone disorders. Based on these findings, gaining a better understanding of the molecular mechanisms involved in cartilage development is extremely important. This knowledge can help guide clinical interventions and preventive strategies to fight bone diseases and improve overall human health.

Calcium-activated nucleotidase 1 (Cant1) is part of the apyrase family and shares similarities with apyrase sequences found in the saliva of hematophagous arthropods [5]. These enzymes break down extracellular nucleotides [e.g., adenosine triphosphate (ATP) and adenosine diphosphate (ADP)] to produce an anti-hemostatic effect [5,6]. While the apyrase function in hematophagous arthropods is well understood, the role of Cant1 in vertebrates is still not fully defined. Current research shows that Cant1 has various biological activities depending on the cell type and tissue location (such as chondrocytes [6], kidney [7], heart [8], and prostate [9]). Importantly, Cant1 is crucial for skeletal development, especially in chondrogenesis. Case reports from different populations (Austria [10], France [11], India [12], Saudi Arabia [13], Turkey [14], United Kingdom [15]) have found Cant1 mutations in individuals with Desbuquois dysplasia (DBQD), a severe skeletal disorder, emphasizing the profound effect of Cant1 mutations on endochondral ossification. Additionally, Cant1 has been linked to the development of scoliosis through multiple pathways, including methylation, immune responses, inflammatory factors, and plasma metabolites [16]. Mutations in Cant1 lead to loss of function, and the resulting cartilage development issues are closely tied to its role in controlling ECM biosynthesis [17,18]. Notably, these skeletal features associated with the disorder can be effectively mimicked in animal models through Cant1 knockout [6,19]. Overall, these findings highlight the vital role of Cant1 in cartilage formation and ECM biosynthesis. However, the molecular mechanisms and downstream signaling pathways through which Cant1 influences vertebrate skeletal abnormalities remain uncertain.

Wingless-like (Wnt) glycoproteins are a family of secreted proteins, with more than 19 members, and are insoluble due to their lipid side chains [20]. The reduced solubility of Wnt ligands contributes to their restricted diffusion and the formation of concentration gradients, which are crucial for influencing neighboring cell behavior [20,21]. β-Catenin is a subunit of the cadherin–catenin complex and is regarded as a key biomarker for activating the canonical Wnt/β-Catenin signaling pathway. It also acts as a co-activator by binding to the T cell factor (TCF)/lymphoid enhancer factor (LEF) transcription factor family, thereby initiating the transcription of downstream target genes [22]. The canonical Wnt/β-Catenin signaling pathway plays a vital role in skeletal formation, homeostasis, and disease development, and has been extensively reviewed in the literature and further supported by our previous findings [23,24]. Given the similar roles of Cant1 and the canonical Wnt/β-Catenin signaling pathway in chondrocyte development and ECM biosynthesis, we are interested in exploring the regulatory relationship between these 2 pathways. Unfortunately, few reports explain the relationship between Cant1 and the canonical Wnt/β-Catenin signaling pathway in chondrocytes.

A central challenge in skeletal developmental biology is understanding how metabolic demand and ECM synthesis are coordinated during rapid chondrogenesis [25]. The broiler chicken, which develops tibial dyschondroplasia (TD) under intensified growth conditions, provides a compelling model for such studies. Its extreme growth rate amplifies metabolic stress and reveals vulnerabilities in ECM biosynthesis and chondrocyte differentiation [26]. Building on this naturally predisposed model, we induced skeletal disruption and altered Cant1 expression in growth plate chondrocytes by knockdown and overexpression. We hypothesize that Cant1 plays a key role in regulating ECM biosynthesis within the growth plate cartilage. This study aims to uncover the molecular mechanisms by which Cant1 influences vertebrate skeletal development, thereby providing a comprehensive understanding of the processes that govern skeletal formation and disease. Our findings are expected to lay the groundwork for developing preventive strategies against human skeletal disorders.

## Results

### Thiram-induced TD demonstrates typical lesions in the tibial growth plate cartilage

**Fig. 1.**
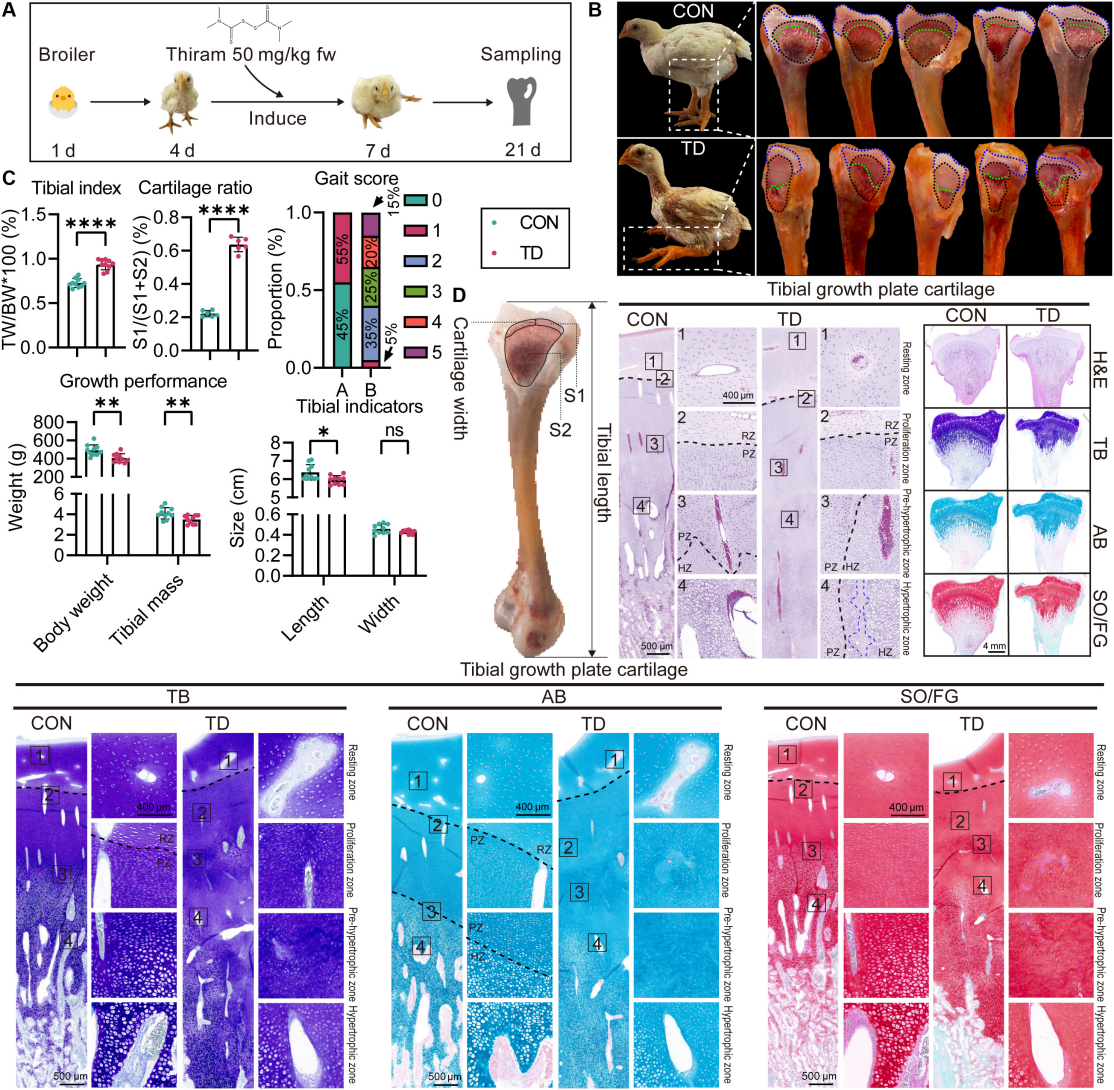
Evaluation of TD models induced by thiram. (A) Illustration of TD model establishment. (B) Gross observation of CON and TD tibias (the blue and black dashed lines indicate the section of the tibial epiphysis, and the green dashed line represents the junction between the growth plate and the bone marrow). (C) Gait score evaluation and quantitative analysis of tibial index, cartilage ratio of the tibial metaphysis, body weight and tibial mass, and tibial length and width between the CON and TD groups. (D) H&E, TB, AB, and SO/FG staining of CON and TD tibias (scale bar shown in the digital diagrams; RZ, resting zone; PZ, proliferative zone; HZ, hypertrophic zone). ns *P* > 0.05, *P < 0.05, **P < 0.01, ***P < 0.001 versus CON group; the same below.

 The thiram-induced TD was caused by dietary intervention, as shown in Fig. 1A. Key symptoms in the TD group included locomotor dysfunction and metaphyseal emboli in the tibial growth plate, compared with the CON group (Fig. 1B). Additionally, the tibial lesions showed significant differences in clinical parameters: The TD group had reduced body weight and tibial length, increased tibial index, and impaired gait score, indicating growth inhibition and abnormal bone development in the TD group (Fig. 1C) (*P* < 0.05). Moreover, histopathological analysis revealed damage to tibial cartilage in the TD group. Evaluation of hematoxylin and eosin (H&E)-, toluidine blue (TB)-, Alcian blue (AB)-, and Safranin O/Fast Green (SO/FG)-stained sections showed distinct pathological changes in 4 regions of interest (ROI) of the growth plate (Fig. 1D). In the resting zone (RZ), chondrocytes were spread throughout the cartilage and surrounded by ECM. TB staining showed a reduction in ECM content of RZ chondrocytes near the proliferation zone (PZ) in the TD group, as indicated by lighter TB staining compared to the CON group. The PZ had rapidly dividing chondrocytes arranged in columns, but TD chondrocytes in the PZ displayed a disordered pattern due to abnormal apoptosis, which released intracellular GAGs and caused varying staining of TB, AB, and SO in the lesion. The pre-hypertrophic zone (PHZ) showed differentiation arrest in the TD group. CON chondrocytes gradually increased in size, while TD chondrocytes remained immature, indicating a failure to begin hypertrophy. The hypertrophic zone (HZ) in the TD group showed severe maturation defects compared to the CON group, marked by incomplete formation of the bone marrow cavity (blue line) and abnormal ECM remodeling. Overall, thiram-induced TD caused significant abnormalities in tibial development. Since TD lesions are closely related to cartilage growth and ECM homeostasis, we next examined the expression of key ECM synthesis factors and components of the Wnt/β-Catenin signaling pathway.

### Suppression of Cant1 and the canonical Wnt/β-Catenin signaling pathway is involved in the thiram-induced TD, impairing the biosynthesis of ECM

**Fig. 2.**
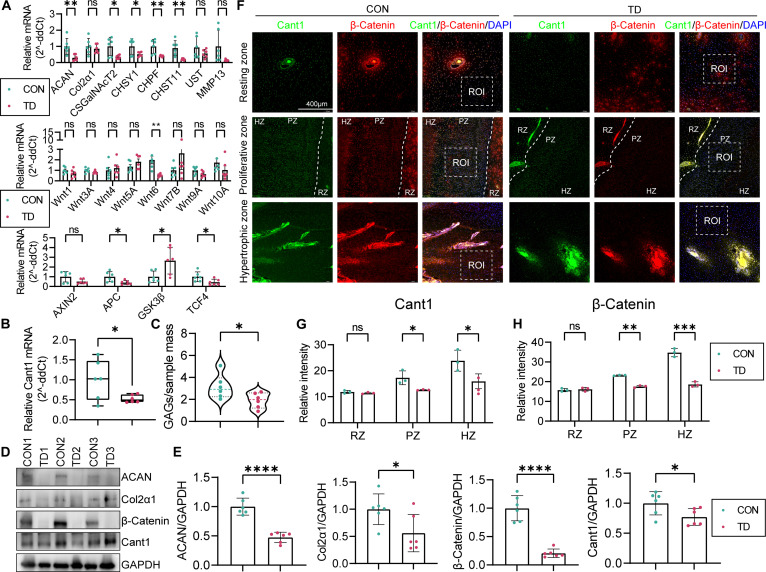
Mechanism of ECM biosynthesis disorders and abnormal Wnt/β-Catenin signaling pathway transduction in TD models. (A) mRNA levels of ECM biosynthesis factors and canonical Wnt/β-Catenin signaling pathway components. (B) mRNA levels of Cant1. (C) Quantitative analysis of GAG content. (D) Protein blots. (E) Protein levels of ACAN, Col2α1, β-Catenin, and Cant1. (F) Immunofluorescence digital images of CON tibia and TD tibia. (G and H) Relative fluorescence intensity of Cant1 and β-Catenin in ROI of CON tibia and TD tibia.

 During skeletal development, cartilage formation is tightly regulated. Disruptions in ECM biosynthesis and Wnt/β-Catenin signaling can cause defects in endochondral ossification. Genetic mutations or Cant1 knockouts that affect ECM biosynthesis lead to chondrodysplasia. To examine the link between Cant1 and TD, and whether ECM biosynthesis and canonical Wnt/β-Catenin signaling pathway activity in the TD tibial growth plate cartilage are affected, the mRNA and protein levels of factors related to ECM biosynthesis and the Wnt/β-Catenin pathway were measured using reverse transcription quantitative polymerase chain reaction (RT-qPCR), Western blot, and immunofluorescence staining. Results showed that the mRNA levels of Cant1, ECM biosynthesis factors (ACAN, CSGalNAcT2, CHSY1, CHPF, and CHST11), and canonical Wnt/β-Catenin pathway factors (Wnt6, APC, and TCF4) were significantly lower in TD models compared to the CON group (*P* < 0.05) (Fig. 2A and B). Conversely, glycogen synthase kinase 3β (GSK3β) levels were significantly higher in the TD group (*P* < 0.05) (Fig. 2A and B). Additionally, the GAG/DNA ratio in tibial growth plate cartilage was significantly reduced in the TD group compared to the CON group (*P* < 0.05) (Fig. 2C). Protein levels of ACAN, Col2α1, β-Catenin, and Cant1 were also notably decreased in the TD group (Fig. 2D and E). Localization analysis revealed that the suppression of Cant1 and β-Catenin protein expression was predominantly observed in the proliferative zone (PZ) and HZ when compared to the CON group (*P* < 0.05) (Fig. 2F to H). These findings suggest that down-regulation of ECM biosynthesis genes and the canonical Wnt/β-Catenin signaling pathway impair ECM production and contribute to TD development.

To validate in vivo findings and establish an in vitro cellular model, we treated TD cells with thiram (Fig. S1A). As expected, intracellular GAG content in TD cells was significantly lower than in CON cells (Fig. S1B). To further confirm consistency between the in vivo and in vitro models, RT-qPCR and Western blot analyses of those markers were performed on the cells. The results showed that Cant1, canonical Wnt/β-Catenin signaling pathway factors (Wnt6, GSK3β, TCF4, and β-Catenin), and ECM biosynthesis factors (ACAN, Col2α1, CHSY1, and CHPF) exhibited the same expression trends in TD cells as in the in vitro model, while the TD group showed down-regulation of APC mRNA, with significant differences compared to CON cells (*P* < 0.05) (Fig. S1C and D). More importantly, the TOPFlash/FOPFlash assay revealed that canonical Wnt/β-Catenin signaling was suppressed due to decreased nuclear entry of β-Catenin in TD cells (Fig. S1E), leading to abnormal transcription of downstream factors. Similarly, the protein levels of ACAN, Col2α1, β-Catenin, and Cant1 showed the same expression trends in TD cells as in the in vitro model, with significant differences compared to CON cells (*P* < 0.05) (Fig. S1F and G). Overall, these results demonstrate a key role for Cant1 and the canonical Wnt/β-Catenin signaling pathway in TD development, which is linked to abnormal ECM biosynthesis.

### Cant1 mediates the biosynthesis of GAG in chondrocytes, associated with the involvement of the canonical Wnt/β-Catenin signaling pathway

**Fig. 3.**
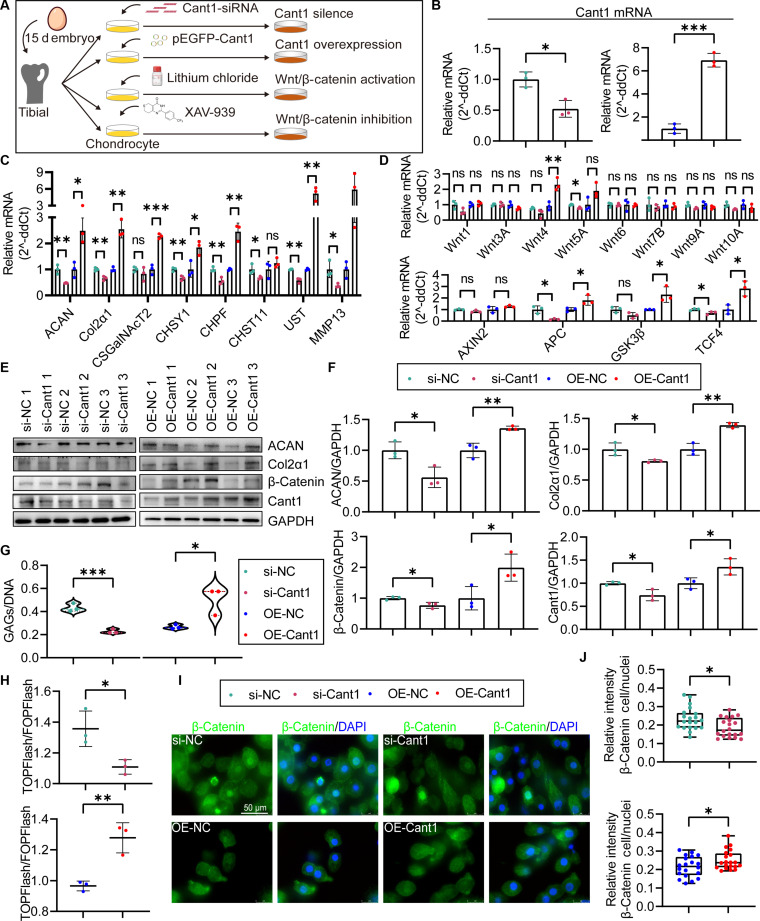
Regulation of Cant1 on ECM biosynthesis and canonical Wnt/β-Catenin signaling pathway activity in chondrocytes. (A) Illustration of Cant1 inhibition/overexpression and canonical Wnt/β-Catenin signaling pathway activation/inhibition in chondrocytes. (B) mRNA levels of Cant1. (C) mRNA levels of ECM biosynthesis factors. (D) mRNA levels of Wnt/β-Catenin signaling pathway components. (E) Protein blots. (F) Protein levels of ACAN, Col2α1, Cant1, and β-Catenin. (G) Quantitative analysis of GAG content. (H) Wnt/β-Catenin transcriptional activity (TOP/FOPFLASH ratio). (I and J) Relative β-Catenin fluorescence intensity in CON and TD chondrocytes.

To directly investigate the role of Cant1 in chondrocytes and its relationship with the canonical Wnt/β-Catenin signaling pathway, we generated Cant1 expression-inhibited and overexpressing tibial growth plate chondrocytes using transient transfection (Fig. 3A). The mRNA levels of Cant1 in si-Cant1 and OE-Cant1 cells were validated by RT-qPCR (*P* < 0.05) (Fig. 3B). The mRNA levels of ECM biosynthesis factors ACAN, Col2α1, CHSY1, CHPF, and UST were significantly altered, with expression patterns consistent with Cant1 levels in the si-Cant1/OE-Cant1 cell models (*P* < 0.05) (Fig. 3C). Furthermore, the canonical Wnt/β-Catenin signaling pathway factors APC and TCF4 were significantly decreased or increased following Cant1 inhibition or overexpression (*P* < 0.05) (Fig. 3D). However, Cant1 overexpression elevated the expression of Wnt4 and GSK3β, while Cant1 inhibition reduced Wnt5A expression (*P* < 0.05) (Fig. 3D). Western blot analyses showed that decreases or increases in Cant1 expression significantly reduced or elevated the protein levels of ACAN, Col2α1, β-Catenin, and Cant1 (*P* < 0.05) (Fig. 3E and F). Additionally, the GAG/DNA ratio indicated that Cant1 positively regulates intracellular ECM biosynthesis, evidenced by significantly reduced GAG content in si-Cant1 cells and increased GAG content in OE-Cant1 cells compared to NC cells (*P* < 0.05) (Fig. 3G), suggesting that Cant1 inhibition impairs, whereas its overexpression enhances, ECM biosynthesis in chondrocytes. To further validate these results, TOPFlash/FOPFlash assays and immunofluorescence analyses demonstrated that decreased or increased Cant1 expression significantly suppressed or promoted β-Catenin nuclear translocation, and reduced or elevated β-Catenin-mediated nuclear transcription (*P* < 0.05) (Fig. 3H to J), confirming that Cant1 positively regulates the canonical Wnt/β-Catenin signaling pathway in chondrocytes.

To explore the connection between the canonical Wnt/β-Catenin signaling pathway and ECM biosynthesis, we measured ECM production in cells where this pathway was either activated or inhibited using the GAG/DNA ratio assay, RT-qPCR, and Western blot. Immunofluorescence and Western blot confirmed successful pathway activation/inhibition (Fig. S2A to D). However, Cant1 protein levels remained unchanged in both groups (Fig. S2C and D). Western blot showed that ACAN and Col2α1 protein levels significantly increased with pathway activation (*P* < 0.05) (Fig. S2C and D). Interestingly, activating Wnt/β-Catenin signaling did not significantly change mRNA levels of ECM factors like ACAN, CHSY1, CHPF, CHST11, and UST (*P* > 0.05), but it did significantly decrease Col2α1, CSGalNAcT2, and MMP13 expression (*P* < 0.05) (Fig. S2E). The GAG/DNA ratio remained similar to that of the CON group (*P* > 0.05) (Fig. S2F), indicating that basal Wnt activity supports ECM biosynthesis, but enhancing it alone is not sufficient without Cant1’s role. Conversely, inhibiting Wnt/β-Catenin signaling markedly reduced ACAN and Col2α1 protein levels (*P* < 0.05) (Fig. S2C and D), decreased mRNA levels of ECM-related factors, including ACAN, Col2α1, CSGalNAcT2, CHSY1, CHPF, CHST11, and UST, and increased MMP13 expression (*P* < 0.05) (Fig. S2E). It also significantly lowered the GAG/DNA ratio compared with dimethyl sulfoxide controls (*P* < 0.001) (Fig. S2F), suggesting that inhibiting this pathway hampers ECM biosynthesis in tibial growth plate chondrocytes. Overall, these results show that the canonical Wnt/β-Catenin signaling pathway governs ECM biosynthesis in tibial growth plate chondrocytes.

### Cant1 promotes chondrocyte ECM biosynthesis by mediating β-Catenin nuclear translocation

**Fig. 4.**
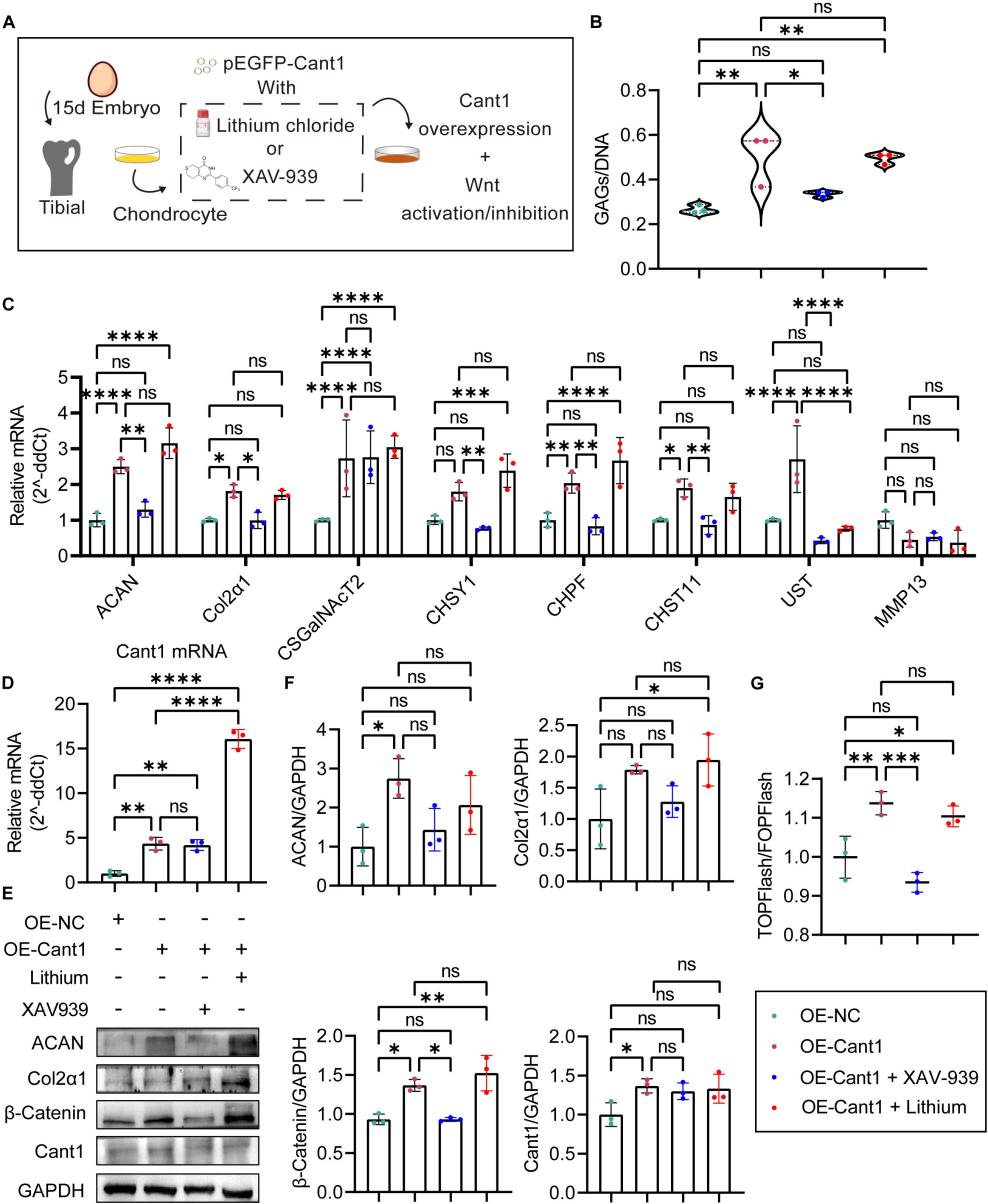
The inhibition of the canonical Wnt/β-Catenin signaling pathway down-regulates ECM biosynthesis promoted by Cant1 overexpression. (A) Illustration of the cell model for Cant1 overexpression combined with activation or inhibition of the canonical Wnt/β-Catenin signaling pathway. (B) Quantitative analysis of GAG content. (C) mRNA levels of ECM biosynthesis factors. (D) mRNA levels of Cant1. (E) Protein blots. (F) Protein levels of ACAN, Col2α1, Cant1, and β-Catenin. (G) Wnt/β-Catenin transcriptional activity (TOP/FOPFLASH ratio).

A direct role for Cant1 in regulating ECM biosynthesis via the canonical Wnt/β-Catenin signaling pathway has not been reported, and our findings provide evidence for this novel mechanism. To further explore the connection between Cant1 and the canonical Wnt/β-Catenin signaling pathway, agonists and inhibitors of this pathway were used in OE-Cant1 cells (Fig. 4A). The results showed that inhibiting the canonical Wnt/β-Catenin signaling pathway reversed the GAG content increase caused by Cant1 overexpression (*P* < 0.05), while activating the pathway did not further increase GAG levels (*P* > 0.05) (Fig. 4B). Similarly, pathway inhibition decreased the mRNA levels of ECM biosynthesis factors ACAN, Col2α1, CHSY1, CHST11, and UST in OE-Cant1 cells, and pathway activation did not further increase these gene expressions (Fig. 4C). Interestingly, pathway activation significantly increased Cant1 mRNA levels (*P* < 0.0001) (Fig. 4D), but did not affect protein levels (*P* > 0.05) (Fig. 4E and F). Moreover, pathway inhibition reduced the protein levels of ACAN, Col2α1, and β-Catenin (Fig. 4E and F), and decreasing β-Catenin protein impaired its transcriptional activity in OE-Cant1 cells, with no additional effect observed from pathway activation (*P* > 0.05) (Fig. 4G). These findings suggest that Cant1 regulates ECM biosynthesis through the canonical Wnt/β-Catenin signaling pathway, and pathway inhibition diminishes Cant1-mediated ECM biosynthesis.

**Fig. 5.**
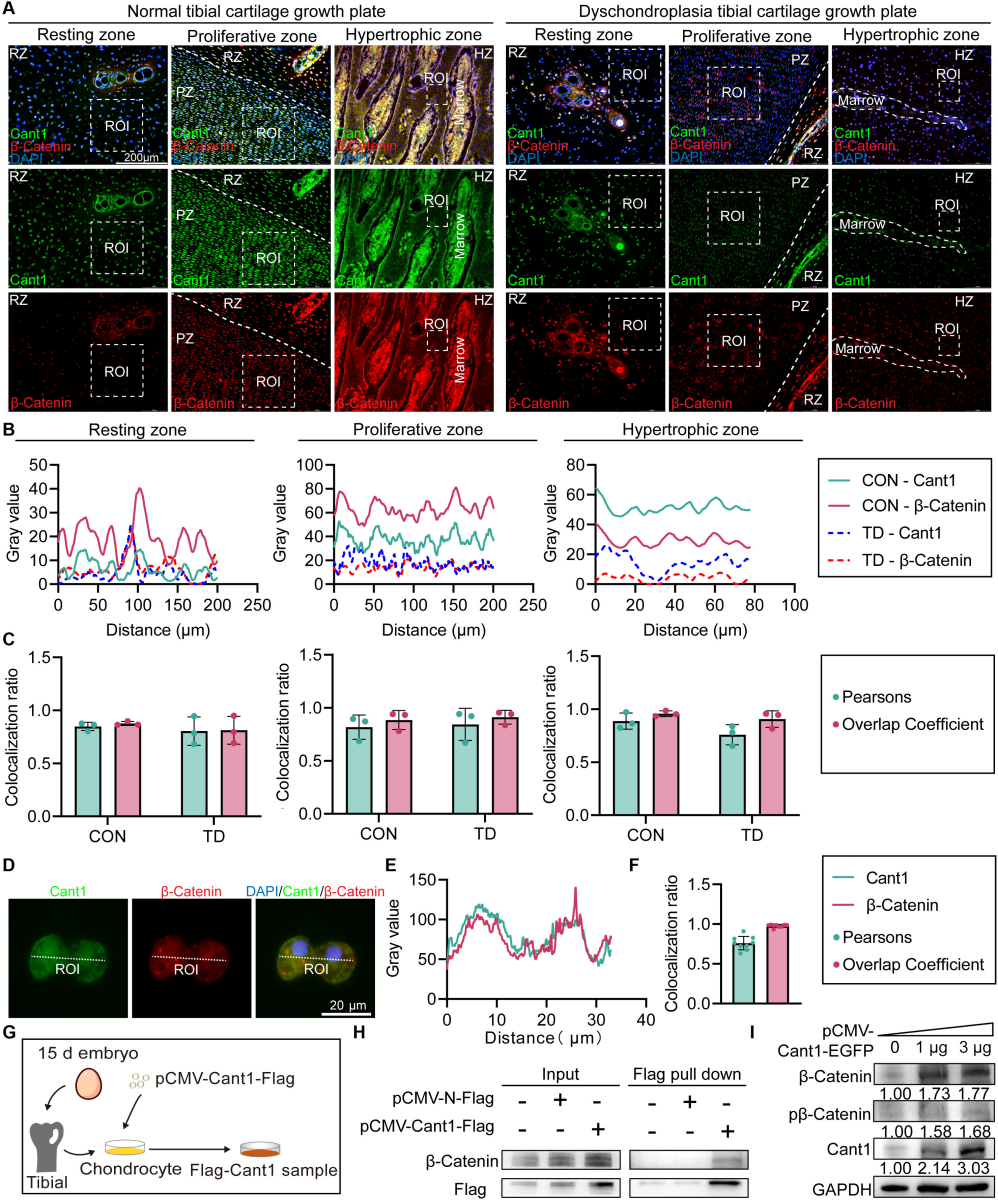
Analysis of the interaction between Cant1 and β-Catenin. (A) Digital immunofluorescence images of CON tibia and TD tibia. (B) Plot profile of Cant1 and β-Catenin fluorescence intensity in ROI of RZ, PZ, and HZ. (C) Colocalization analysis of Cant1 and β-Catenin fluorescence intensity in ROI of RZ, PZ, and HZ. (D) Digital immunofluorescence images of chondrocytes. (E) Plot profile of Cant1 and β-Catenin fluorescence intensity in ROI of chondrocytes. (F) Colocalization analysis of Cant1 and β-Catenin fluorescence intensity in ROI of chondrocytes. (G) Illustration of cell model construction for Co-IP assay. (H) Co-IP of Cant1 and β-Catenin proteins in chondrocytes. (I) Protein levels of Cant1 and β-Catenin.

To better understand the mechanism of Cant1’s role in the canonical Wnt/β-Catenin signaling pathway, we examined the interaction between Cant1 and β-Catenin both in vivo and in vitro. Colocalization analysis of Cant1 and β-Catenin in various regions of tibial growth plate cartilage was conducted (Fig. 5A). The results revealed that the plot profiles of Cant1 and β-Catenin protein levels in the ROI of PZ and HZ showed similar expression patterns, whereas in RZ, the expression levels were too weak to discern these trends (Fig. 5B). However, Pearson’s correlation (*r* > 0.5) and overlap coefficient confirmed significant colocalization across all regions (Fig. 5C). Similarly, the plot profiles of Cant1 and β-Catenin in cellular ROIs exhibited comparable expression patterns, with Pearson’s (*r* > 0.5) and overlap coefficient values indicating significant colocalization (Fig. 5D to F). Additionally, we created pCMV-Cant1-Flag vectors to verify the interaction between Cant1 and β-Catenin in a coimmunoprecipitation (Co-IP) assay (Fig. 5G). The results demonstrated that the Cant1–β-Catenin protein complex was successfully pulled down and detected, confirming an endogenous binding between Cant1 and β-Catenin in chondrocytes (Fig. 5H). To explore the interaction mechanism further, we performed transient transfection with increasing doses of the pCMV-Cant1-EGFP vector. We observed that both total and phosphorylated β-Catenin protein levels increased in a dose-dependent manner with Cant1 expression. This suggests that Cant1 does not promote β-Catenin expression by inhibiting its phosphorylation but likely through other mechanisms that facilitate β-Catenin accumulation (Fig. 5I). In conclusion, these findings demonstrate that Cant1 naturally interacts with β-Catenin and enhances Wnt/β-Catenin signaling by increasing β-Catenin protein levels, thereby further activating transcription of downstream genes involved in ECM biosynthesis.

### Cant1/β-Catenin axis-mediated CHSY1 transcription via TCF4 interacted with CHSY1 promoter region, regulating ECM biosynthesis

**Fig. 6.**
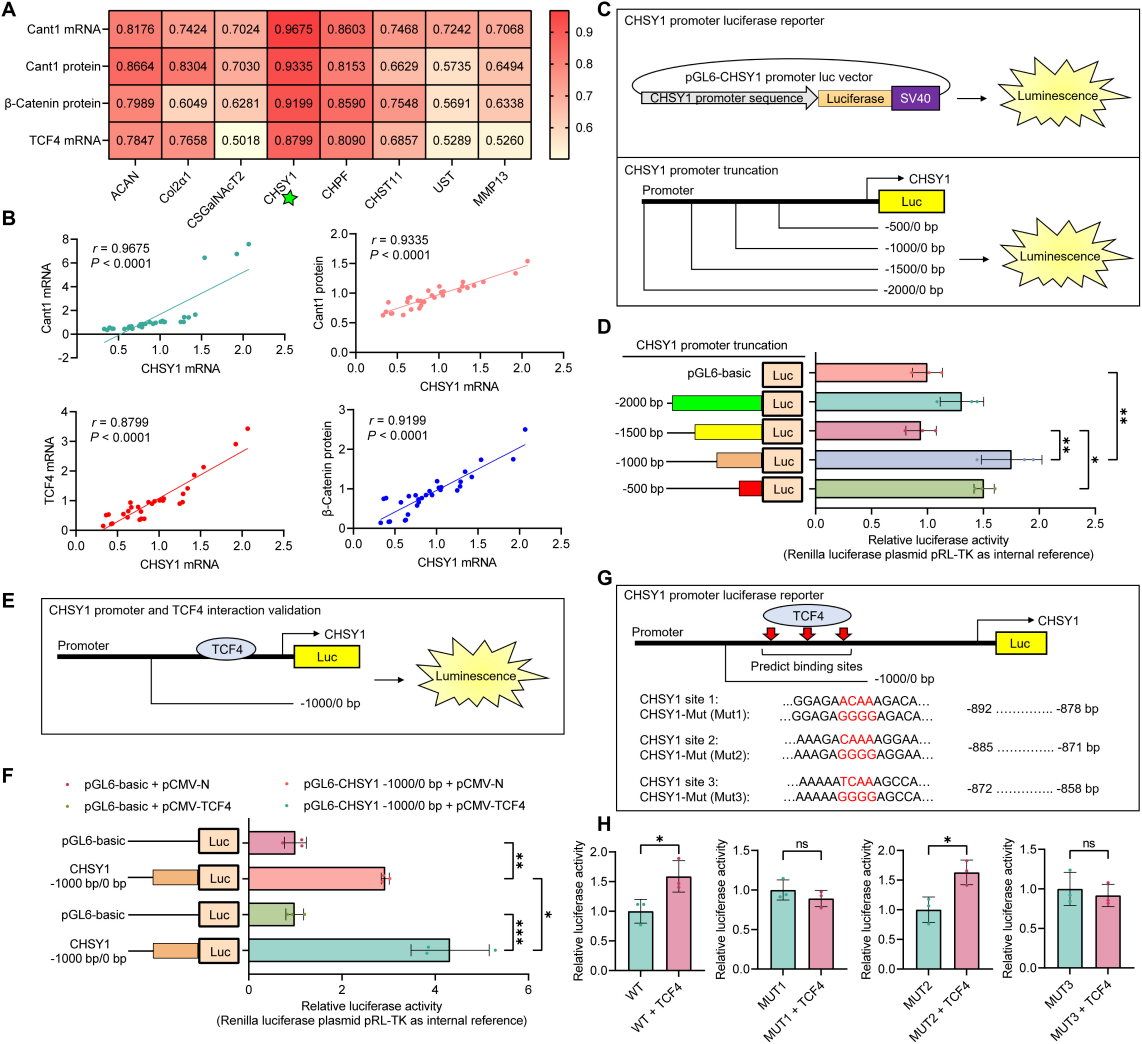
Canonical Wnt/β-Catenin signaling pathway transcription factor TCF4 transcriptionally activates CHSY1 expression in chondrocytes. (A and B) Correlation analysis of ECM biosynthesis factors with key factor expression levels. (C) Illustration of the CHSY1 promoter luciferase reporter constructs used in the study. (D) Luciferase activity of the full-length and truncated CHSY1 promoter. (E and F) Luciferase activity of the CHSY1 promoter in cells transfected with a basic plasmid or a TCF4 overexpression plasmid. (G) Potential TCF4-binding sites in the CHSY1 promoter sequence. (H) Luciferase activity of the CHSY1 promoter (WT, MUT1, MUT2, and MUT3) in cells.

It is well known that TCF4 is a transcription factor. Based on our findings that TCF4 expression correlates with Cant1 levels and functions as a canonical Wnt/β-Catenin signaling effector, we examined its role in regulating the transcription of ECM biosynthesis factors. To explore these connections, we analyzed correlations between ECM biosynthesis factors and Cant1, β-Catenin, and TCF4 using models derived from TD broiler tibias, TD cells, and Cant1 overexpression/inhibition cells. The results showed that all ECM biosynthesis factors had significant correlations (*r* > 0.5, *P* < 0.05) with Cant1, β-Catenin, and TCF4 (Fig. 6A). Notably, CHSY1 mRNA expression displayed the highest correlation coefficients with all 3 regulatory factors (*r* > 0.8, *P* < 0.0001), and in the Cant1 overexpression/inhibition cells, the correlation coefficient between TCF4 and CHSY1 changed in sync with Cant1 levels (Fig. 6B). Given the strong correlation between CHSY1 and Cant1/β-Catenin/TCF4, we hypothesize that TCF4 directly regulates CHSY1 transcription. To clarify this regulatory mechanism, we investigated the interaction between TCF4 and CHSY1. Various truncations of the CHSY1 promoter region and a luciferase reporter assay identified the core promoter region (from −1,000 to 0), which was then cloned (Fig. 6C and D). The luciferase reporter assay with the truncated CHSY1 promoter (−1,000 to 0) further confirmed TCF4 binding to the CHSY1 promoter (Fig. 6E and F). Next, we identified potential TCF4-binding sites in the CHSY1 promoter (site 1: 892 to 878, site 2: 885 to 871, and site 3: 872 to 858) using AnimalTFDB4 (https://guolab.wchscu.cn/AnimalTFDB4/#/), and mutating these sites showed that MUT1 and MUT3 suppressed TCF4-induced luciferase activity of the CHSY1 promoter (Fig. 6G and H).

Next, to clarify how Cant1 specifically regulates downstream signaling through CHSY1 to influence ECM biosynthesis, we established combined cellular models of Cant1 inhibition or overexpression along with CHSY1 inhibition (Fig. S3A). The results showed that inhibiting CHSY1 significantly reduced GAG content in chondrocytes, similar to the effect of Cant1 inhibition, compared to the NC group (*P* < 0.05) (Fig. S3B). However, simultaneous inhibition of both CHSY1 and Cant1 did not cause a further significant decrease in GAG content. Additionally, CHSY1 inhibition prevented the increase in GAG content caused by Cant1 overexpression (Fig. S3B). These findings indicate that CHSY1 is involved in Cant1-mediated ECM biosynthesis in chondrocytes. Importantly, CHSY1 inhibition did not affect Cant1 mRNA or protein levels, whereas Cant1 positively regulated CHSY1 mRNA levels, suggesting that Cant1 acts upstream of CHSY1 and partly influences chondrocyte ECM biosynthesis through it (Fig. S3C to F). Further experiments showed that inhibiting CHSY1 significantly decreased the protein levels of ACAN and Col2α1, and combining Cant1 and CHSY1 inhibition strengthened this inhibitory effect (*P* < 0.05) (Fig. S3E and F). Similarly, CHSY1 inhibition reduced the up-regulation of ACAN and Col2α1 protein levels caused by Cant1 overexpression (Fig. S3E and F). Similar changes were observed at the mRNA level as well. Double inhibition of Cant1 and CHSY1 further decreased the mRNA levels of ACAN, Col2α1, and CHPF (*P* < 0.05) (Fig. S3G). Under conditions of Cant1 overexpression, CHSY1 inhibition countered the increase in mRNA expression of ACAN, Col2α1, CSGalNAcT2, CHPF, CHST11, and UST caused by Cant1 (*P* < 0.05) (Fig. S3H). Overall, these findings show that the Cant1/β-Catenin–TCF4 pathway, at least partly, promotes GAG biosynthesis by transcriptionally activating CHSY1 expression in cells.

## Discussion

This study uncovers a previously unknown signaling axis, the Cant1/β-Catenin/TCF4–CHSY1 pathway, as a crucial regulator of ECM biosynthesis in skeletal development. The main novelty of this work is clarifying Cant1 as a direct upstream regulator of β-Catenin and connecting it to the Wnt/β-Catenin signaling pathway. Additionally, it reveals how this axis influences ECM biosynthesis by activating the transcription of the key gene CHSY1 through the TCF4 transcription factor. These findings offer a new explanation for the development of Cant1-related skeletal disorders, highlighting the dysregulation of this pathway as a central factor in their pathology.

Broilers serve as a highly relevant model for studying human skeletal disorders because of their rapid growth rates, conserved regulatory pathways in cartilage development, and their responsiveness to genetic and environmental interventions [27–29]. Their exceptional growth performance intensifies metabolic and ECM homeostasis imbalances, making them especially useful for uncovering the mechanisms behind skeletal dysplasia. In this study, the TD model was used due to its remarkable similarity to Cant1 dysfunction-related skeletal abnormalities in humans, particularly regarding pathological phenotypes and ECM metabolic issues [6,30]. Our focus was on understanding how Cant1 influences chondrogenesis, especially its downstream signaling effectors and their interactions. The broiler model thus acts not only as a disease model but also as an effective platform for validating potential therapies, bridging the gap between mechanistic research and translational applications for skeletal disorders.

The multiple biological functions of Cant1 across tissues and cell types have been reported in previous studies, including nucleotide hydrolysis, ECM biosynthesis, cell proliferation, cell invasion, and molecular signaling [6,7]. In particular, Cant1 plays a key role in regulating ECM biosynthesis, and mutation or depletion of this gene can lead to protein dysfunction and aberrant ECM biosynthesis. Cant1-knockout mice exhibit skeletal disorders similar to those in DBQD1 patients, along with reduced GAG content and altered ECM composition in the growth plate cartilage, resulting in impaired chondrocyte proliferation and hypertrophic differentiation [6,19]. Furthermore, studies in mouse chondrocyte models suggest that Cant1 deficiency disrupts ECM biosynthesis through mechanisms involving morphological alterations of the endoplasmic reticulum (ER), independent of canonical ER stress pathways [6]. Collectively, these findings underscore the conserved role of Cant1 in GAG synthesis and skeletal development. Although mutations in Cant1 have been reported in human skeletal disorders, a comprehensive understanding of the biochemical, molecular, and cellular events mediated by Cant1 in cartilage and bone remains incomplete, as bone tissue biopsies from individuals with Cant1 mutations are rarely available.

The canonical Wnt/β-Catenin signaling pathway is a highly conserved and critically important intracellular signaling pathway. Among these processes, the stable buildup and nuclear translocation of β-Catenin within the cell act as the key regulator determining the expression fate of downstream target genes [31]. The Wnt/β-Catenin pathway is a vital regulator of essential biological processes, including embryonic development, tissue homeostasis, cellular proliferation and differentiation, and stem cell self-renewal. Moreover, dysregulation of this pathway is linked to the development of skeletal disorders [31]. Numerous gain- and loss-of-function studies in vivo have shown that both excessive activation and deficiency of pathway signaling can result in skeletal dysplasia and even early death [24]. In patients with osteoarthritis, both increased and decreased Wnt/β-Catenin signaling contribute to cartilage breakdown, worsen inflammation, and accelerate disease progression [32–34]. Additionally, reduced expression of nonphosphorylated β-Catenin in bone tissue has been associated with osteoporosis and a higher risk of fractures in individuals with type 2 diabetes and obesity [35]. Likewise, mutations in genes encoding negative regulators of β-Catenin can also cause skeletal dysplasia by boosting β-Catenin activity [36]. Overall, these findings highlight that the canonical Wnt/β-Catenin pathway plays a crucial role in the development of a wide range of human skeletal diseases.

In this context, we found that TD models exhibited reduced Cant1 mRNA and protein levels alongside severe tibial damage. Additionally, the dysregulation of key factors such as Wnt6, APC, GSK3β, and TCF4 (also called TCF7L2) in the Wnt/β-Catenin signaling pathway, along with the significant reduction in β-Catenin protein levels, indicates that inhibition of this pathway causes signal transduction blockade and worsens the development of TD. This finding aligns with previous studies [23,30]. Interestingly, we observed down-regulation of APC mRNA in the in vivo model, while its expression was up-regulated in the in vitro model. As a core part of the canonical Wnt/β-Catenin destruction complex, APC plays a dual role in signal transduction: (a) Its association with AXIN and GSK3 promotes β-Catenin degradation, facilitates nuclear export, and opposes β-Catenin/TCF-mediated transcription; (b) it can also regulate AXIN stability or enhance β-Catenin accumulation to promote signaling [37,38]. These contrasting APC mRNA expression patterns emphasize the complexity of canonical Wnt/β-Catenin pathway regulation in TD cartilage. Furthermore, immunofluorescence analysis of tissue sections showed a significant decrease in both Cant1 and β-Catenin protein levels in the PZ and HZ of the tibial growth plate in TD models. This result supports the known role of the Wnt/β-Catenin pathway in promoting chondrocyte hypertrophy and maturation, as increased β-Catenin activity is typically seen in specific zones of the growth plate [39,40]. Likewise, TD lesions mainly damage chondrocytes in the transition zone from the PZ to the HZ of the growth plate. This vulnerability likely results from impaired differentiation processes during their high proliferative activity [41]. In sum, the notable reduction of both Cant1 and β-Catenin proteins in these regions highlights the critical role of these signaling molecules in TD lesion development. However, the regulatory relationship between these factors and their role in worsening TD lesions still needs to be clarified.

The cartilage, composed of chondrocytes and ECM produced by these cells, has its physical function of joints and its biochemical properties mainly dictated by the integrity of the ECM [42]. The proteoglycan, composed of sulfated GAGs and core proteins, forms noncovalent associations with components such as hyaluronic acid and collagen fibers, creating an environment that supports the survival and growth of chondrocytes [42]. Previous studies have used increases in ECM biosynthesis as a marker of chondrocyte differentiation, and disruptions in ECM biosynthesis within cartilage ECM can result in cartilage dysplasia [43]. Here, we observed that GAG content in tibial chondrocytes from TD models was significantly reduced, aligning with previous reports [44]. Unlike HA, which is synthesized directly from the plasma membrane and secreted into the pericellular zone without modification, the synthesis of sulfated GAG involves multiple enzymatic steps in the Golgi apparatus. In brief, under the action of glycosyltransferases (such as CSGaLNAcT2, CHSY1, and CHPF), the initial GAG skeleton is assembled in the Golgi. Subsequently, GlcA and GlcNAc residues are added and extended into a sugar chain through the catalytic activity of glycosyltransferases. The disaccharide units are then modified by sulfation, performed by carbohydrate sulfotransferases (such as CHST11 and UST), to produce the final long-chain structures of GAGs, such as chondroitin sulfate and dermatan sulfate [45]. Additionally, further investigation revealed significantly reduced expression of ECM biosynthesis factors, including ACAN, Col2α1, CSGalNAcT2, CHSY1, CHPF, and CHST11 in tibial chondrocytes of TD models, demonstrating the molecular mechanisms behind impaired ECM biosynthesis in these models.

Although these findings have revealed the expression profiles of Cant1 and β-Catenin and identified the molecular mechanisms underlying impaired ECM biosynthesis in TD models, the regulatory relationship between these factors remains to be further clarified. Therefore, we independently examined the regulatory mechanisms of both Cant1 and the canonical Wnt/β-Catenin signaling pathway on ECM biosynthesis in chondrocytes. As expected, Cant1 positively influences ECM biosynthesis in chondrocytes, as detailed in the Results section. Previous studies suggest that ECM biosynthesis defects caused by Cant1 mutations or deficiencies primarily result from impaired GAG chain elongation and abnormal sulfation, ultimately reducing proteoglycan synthesis [6,46]. Here, we offer mechanistic insights into this defect by exploring its transcriptional regulation. Moreover, we propose that a key mechanism by which Cant1 regulates ECM biosynthesis involves its nucleosidase activity interacting with uridine diphosphate (UDP)-sugars, which are essential substrates for GAG chains, given its highest catalytic activity toward UDP [substrate activity profile: UDP > guanosine diphosphate (GDP) > inosine diphosphate (IDP) >> uridine triphosphate (UTP) > cytidine diphosphate (CDP) = guanosine triphosphate (GTP) = inosine triphosphate (ITP)] [6,19]. However, the functional impact of Cant1 on ECM biosynthesis remains unknown and needs experimental validation in future studies. The canonical Wnt/β-Catenin signaling pathway plays a critical role in regulating skeletal development, longitudinal growth, and tissue homeostasis. This pathway is tightly controlled, as both overactivation and suppression may contribute to the development of bone disorders [47]. Therefore, the pathway’s complexity and potential effects of pharmacological interventions highlight the urgent need for further research. Our findings show that activating the Wnt/β-Catenin pathway with lithium chloride did not enhance the transcription of ECM biosynthesis factors or increase GAG content in chondrocytes. However, we observed an up-regulation of ECM components Col2α1 and ACAN proteins. We suggest that this may be linked to the inhibition of MMP13, as suppression of metalloproteinase activity reduced ECM degradation [48]. Conversely, inhibiting the canonical Wnt/β-Catenin pathway with XAV-939 led to transcriptional dysregulation of ECM biosynthesis factors and significantly reduced intracellular GAG levels, similar to what is observed in TD chondrocytes. Overall, these results clarify the dual regulatory role of the canonical Wnt/β-Catenin pathway in modulating ECM biosynthesis in tibial chondrocytes.

More importantly, understanding the relationship between Cant1 and the canonical Wnt/β-Catenin signaling pathway is crucial for explaining their molecular roles in regulating ECM biosynthesis in chondrocytes. In addition to canonical and noncanonical signaling pathways that promote β-Catenin accumulation and nuclear translocation, many studies have reported interactions between β-Catenin and latent proteins that can enhance or suppress pathway activity [49,50]. Here, we identify Cant1 as a positive regulator of the canonical Wnt/β-Catenin pathway and further show that it acts as an upstream effector; this result aligns with earlier findings in human lung squamous epithelial cell lines [51]. Additionally, our study provides direct molecular evidence of a specific interaction between Cant1 and endogenous β-Catenin, indicating that Cant1 enhances canonical Wnt/β-Catenin signaling. We hypothesized that Cant1 might promote β-Catenin buildup by decreasing its phosphorylation. However, our results showed that Cant1 accumulation does not prevent β-Catenin phosphorylation; instead, it raises β-Catenin levels and promotes its nuclear translocation through other mechanisms. Interestingly, while altering Cant1 primarily affected the expression of core pathway components (such as TCF4 and β-Catenin), its influence on certain Wnt ligands (like Wnt4 and Wnt5A) and on GSK3β varied and did not consistently match Cant1 levels. This suggests that these factors are unlikely to be direct targets of the Cant1/β-Catenin axis but may be part of compensatory mechanisms within the broader Wnt signaling network during Cant1 regulation. Although we revealed a functional link between Cant1 and β-Catenin, key mechanistic details remain unclear. The specific way Cant1 enhances β-Catenin accumulation, whether through protein stabilization, translational control, other modifications, or complex formation, has yet to be determined. Furthermore, Cant1’s regulatory role may extend beyond this direct interaction to include influences on other Wnt components or crosstalk with pathways like TGF-β or BMP. Future studies aim to clarify these mechanisms and map the entire signaling network, providing a more comprehensive understanding of Cant1’s role in skeletal development and maintenance. Notably, the impaired ECM biosynthesis observed in TD models can be attributed to the Cant1–β-Catenin interaction, which modulates canonical Wnt/β-Catenin signaling. This suggests that therapeutic agents targeting this pathway, whether synthetic compounds or food-based bioactive extracts, could have potential in treating TD by modulating this mechanism [30,52,53].

Interestingly, the activity of the canonical Wnt/β-Catenin signaling pathway, modulated by Cant1, affects downstream transcription factors regulated by β-Catenin/TCF4. In canonical Wnt signaling, stabilized β-Catenin translocates from the cytoplasm to the nucleus and binds to LEF1 and TCF transcription factors to activate the transcription of Wnt target genes [31]. Previous studies indicate that TCF4 participates in cellular development, including the regulation of ECM biosynthesis and metabolic processes [54]. In this study, Cant1 inhibition was associated with TD models that showed suppression of TCF4 transcription and impairment of ECM biosynthesis in tibias. Therefore, we hypothesize that inhibiting Cant1 in TD broilers reduces Wnt/β-Catenin signaling activity, thereby decreasing transcription of ECM biosynthesis-related factors mediated by β-Catenin/TCF4. To identify the most relevant downstream effector linking β-Catenin/TCF4 to ECM biosynthesis, we performed correlation analysis and identified CHSY1 as the primary target, with its expression closely linked to the Cant1/β-Catenin/TCF4 axis (*r* > 0. 0.8). Beyond its established role in ECM biosynthesis, CHSY1 is essential for skeletal development and digit formation. Mutations or loss-of-function in CHSY1 is known to cause human syndromic brachydactyly, a form of skeletal dysplasia characterized by hyperostosis, facial dysmorphism, dental anomalies, sensorineural hearing loss, delayed motor and intellectual development, and growth retardation, as documented in previous studies [55–57]. Although CHSY1 is recognized for its critical role in GAG biosynthesis, recent research indicates that its functions extend beyond this canonical activity, including regulation of other ECM-related genes and proteins [58]. This study shows that CHSY1 inhibition alters the expression of ECM biosynthesis-related genes and proteins and impairs GAG synthesis, consistent with previous research [59,60]. The effects of Cant1-mediated ECM biosynthesis, partly dependent on CHSY1, suggest that Cant1 influences chondrocyte ECM homeostasis not only through the Cant1/CHSY1 axis but also likely via additional regulatory mechanisms. Overall, our data clarify CHSY1’s specific role in Cant1-driven regulation of chondrocyte ECM biosynthesis. Importantly, these findings provide evidence for TCF4-mediated transcriptional regulation of CHSY1. This advances our understanding of ECM biosynthesis by revealing a new layer of transcriptional regulation of GAGs. Future studies examining the expression and correlation of CANT1, β-Catenin, and CHSY1 in chondrocytes or cartilage tissue from patients with skeletal disorders will be essential to confirm the translational relevance of this signaling pathway.

## Conclusion

In this study, Cant1 directly binds to β-Catenin and promotes its accumulation, thereby enhancing canonical Wnt/β-Catenin signaling. This activation leads to the transcription of key GAG biosynthetic effectors, especially CHSY1, via TCF4 that maintain cartilage ECM homeostasis. Dysfunction of the Cant1–β-Catenin–TCF4 regulatory axis is a key mechanism underlying TD pathobiology. These findings present new targets for developing precision treatments for cartilage disorders.

## Materials and Methods

### Ethics statement

In this study, all animal experiments were performed following the guidelines approved by the South China Agricultural University Institutional Animal Care and Use Committee (approval number: 2022F045).

### Animals

The Arbor Acres broiler chickens (*n* = 50, 1 d old, weighing 40 ± 5 g) were purchased from a commercial hatchery and housed in the experimental animal center of South China Agricultural University during the experiment. Before starting the experiment, the henhouse was thoroughly cleaned and disinfected with formaldehyde [30]. Additionally, the feed and equipment were subjected to ultraviolet disinfection before being introduced into the henhouse. The broilers were provided with food and water ad libitum, fed twice daily, and maintained under a 12-h light/dark cycle. They were randomly divided into 2 experimental groups for a 21-d period: a CON group receiving a standard diet and a TD group fed a diet supplemented with 50 mg/kg thiram. Thiram was used as a validated TD inducer that disrupts chondrocyte differentiation and growth plate vascularization [53].

Before weighing and sampling, the broilers' walking was observed, and their walking ability was scored on a scale of 0 to 5 (gait score) [61]. The scoring standard shown in Table S1 was used. Twenty broilers from each group were randomly selected and euthanized via cervical dislocation. The tibias from broilers were dissected and measured. Then, some of the tibial growth plate cartilages were collected and stored at −80 °C for measurement, while others were fixed in 4% paraformaldehyde for morphological evaluation.

### Cell culture and treatment

The primary chondrocytes were isolated from the tibial growth plate cartilage of a broiler embryo, as detailed in a previous study [62]. Cells were cultured in Dulbecco’s modified Eagle’s medium (DMEM)/F12 complete medium supplemented with 10% fetal bovine serum (YiLang, China) in a humidified incubator at 37 °C with 5% CO_2_. To establish a consistent model of TD, cells were treated with 10 μM thiram (no. W10363, Shyuanye, China) for 48 h after reaching 80% confluency—a concentration and duration previously confirmed to reliably induce dyschondroplasia-like impairment in chondrocyte function and matrix homeostasis [23,30,62]. For activation or inhibition of the canonical Wnt/β-Catenin signaling pathway, cells were cultured in DMEM/F12 containing 5 mM lithium chloride (no. R30508, Shyuanye, China) or 10 μM XAV-939 (no. HY-15147, MedChemExpress, China) for 48 h. Cells were then washed with phosphate-buffered saline (PBS), quenched in liquid nitrogen, and stored at −80 °C for further analysis.

### GAG/DNA ratio quantification

To measure the intracellular GAGs, cells were digested with a buffer containing 12.5% papain (no. S10011, Shyuanye, China), 1.68% EDTA-Na (no. E909976, Maclin, China), and 0.57% cysteamine hydrochloride (no. C804763, Maclin, China) at 60 °C for 24 h. Then, GAG content was measured using the dimethylmethylene blue (no. D823055, Macklin, China) assay with a chondroitin sulfate standard (no. C875626, Macklin, China). DNA was extracted with a genomic DNA isolation kit (no. Sup-021603, Surbiopure, China), and its quantity was determined using a Nanodrop Spectrophotometer (Thermo Fisher Scientific, USA). The GAG-to-DNA ratio was then calculated [30].

### Gene expression analysis

Total RNA was extracted from cells using TRIzol Reagent (catalog no. 109, Takara, Japan) and purified as described in a previous study (26). The RNA samples were reverse transcribed with a reverse transcription kit (no. RK20429, ABclonal, China). Quantitative PCR was carried out using Universal SYBR Green Fast qPCR Mix (no. RK21203, ABclonal, China) on qTOWER3G (JENA, Germany) with validated primers (Tsingke, China) (Table S2).

### Plasmids and transfection

Cant1 coding sequences, TCF4 coding sequences, and CHSY1 promoter sequences were generated by PCR (no. AS231, TransGen, China) and cloned into BglII and EcoRI, or HindIII and EcoRI, or KpnI and BglII sites (no. 1606/1611/1615/1618, Takara, Japan) of pCMV-N-EGFP (Promega, USA), pCMV-N-Flag (no. D2632, Beyotime, China), and pGL6-TA vector (no. D2105, Beyotime, China) using Seamless Assembly (no. C5891, Clone Smarter, China). The constructs were transformed into *Escherichia coli* DH5α competent cells (no. 9057, Takara, Japan). The small interfering RNA (siRNA) targeting Cant1 (si-Cant1) and CHSY1 (si-CHSY1) was synthesized by GenePharma (GenePharma, China). For Cant1 overexpression/inhibition and CHSY1 inhibition, cells were cultured until 80% confluency and transfected with either pCMV-Cant1-EGFP, pCMV-Cant1-Flag, si-Cant1, si-CHSY1, or si-negative control (si-NC), pCMV-N-EGFP, pCMV-N-Flag, or pGL6-TA empty vector. Transfection was performed using HighGene Plus Transfection Reagent (no. RM09014, ABclonal, China) according to the previous study’s instructions. The primers utilized in this study are provided in Tables S3 and S4.

### Luciferase reporter activity assays

To validate Cant1-regulated function, cells were cultured to 80% confluency and transfected with pCMV-Cant1-EGFP or pCMV-N-EGFP, respectively, along with TOPflash or FOPflash. For TD in vitro, cells were cultured to 80% confluency, transfected with the TOPflash or FOPflash vector, and then incubated with 10 μM thiram. Forty-eight hours after transfection, luciferase activity was assayed using the Firefly Luciferase Reporter Gene Assay Kit (no. RG005, Beyotime, China). TOPflash vector activities were normalized to FOPflash vector activity.

Potential binding sites for TCF4 on the CHSY1 promoter were predicted using AnimalTFDB v4.0 (https://guolab.wchscu.cn/AnimalTFDB4/#/). DNA site mutation was performed according to the manufacturer’s protocol (no. C214, Vazyme, China). The primers used in this study are listed in Table S5. To validate CHSY1 promoter function, cells were cultured until 80% confluency and transfected with pGL6-CHSY1 promoter (full-length, truncated, or site-mutated sequences) or pGL6-TA vector, along with pRL-TK (no. D2760, Beyotime, China) and/or pCMV-TCF4-Flag vector. After 48 h, luciferase activity was measured using the Dual-Lumi Luciferase Assay Kit (no. RG088, Beyotime, China).

### Western blotting

The protocol was carried out according to the instructions in the previous study [63]. Total protein from samples was extracted using radioimmunoprecipitation assay (RIPA) buffer (no. P0013B, Beyotime, China) containing 1 mM phenylmethanesulfonyl fluoride (no. ST2573, Beyotime, China) and denatured with loading buffer (no. CW0027S, CWBio, China). Protein samples were separated by electrophoresis on 12.0% sodium dodecyl sulfate–polyacrylamide gel electrophoresis (SDS-PAGE) gels and transferred to a 0.45-μm polyvinylidene difluoride (PVDF) membrane (no. IPFL00010, Millipore, Germany). Protein bands were blocked in tris–borate–sodium Tween-20 (TBST) with 5% skim milk and then incubated with primary antibodies against Cant1 (no. A6341, ABclonal, China), β-Catenin (no. 66379-1-Ig, Proteintech, China), phospho-β-Catenin (no. AP1076, ABclonal, China), Col2α1 (no. A1520, ABclonal, China), glyceraldehyde-3-phosphate dehydrogenase (GAPDH) (no. 60004-1-1g, Proteintech, China), and ACAN (no. A12045, ABclonal, China). Afterward, samples were incubated with horseradish peroxidase (HRP)-conjugated secondary antibodies (no. CW0103S/CW0102S, CWBio, China). The protein bands were visualized using the ECL Super Kit (no. RM02867, ABclonal, China), and images were captured with a chemiluminescence imaging instrument (Tanon, China).

### Indirect immunofluorescence staining

For tissue sections, the detailed protocol of rehydration, antigen repair, blocking, and antibody incubation has been previously described [64]. For cell fixation, cells were fixed with 4% paraformaldehyde for 30 min. After fixation, 4% paraformaldehyde was replaced with methanol, and cells were incubated for 20 min. Cells were washed with PBS for 5 min, incubated with fluorescence quencher (no. G1221, Servicebio, China) for 30 min, washed again with PBS for 5 min, and blocked with blocking buffer (no. P0260, Beyotime, China) for 15 min. Subsequently, they were incubated with Cant1 (no. A6341, ABclonal, China) and β-Catenin (no. 66379-1-Ig, Proteintech, China) primary antibodies, followed by incubation with CoraLite488 (no. SA00013-2, Proteintech, China) and/or CoraLite Plus 555 (no. RGAR003, Proteintech, China) secondary antibodies. Digital images were captured using a Leica DM4 B (Leica, Germany).

### Histopathological evaluation

The detailed protocol for paraffin embedding, tissue sectioning, and H&E staining (no. G1005, Servicebio, China) in histopathological evaluation has been outlined in the instructions of a previous study [65]. The AB, SO/FG, and TB staining procedures were performed on 5-μm sections from control and TD tibial samples using the AB, SO/FG, and TB cell and tissue staining kits (no. G1027/G1053/G1032, Servicebio, China) according to the manufacturer’s instructions. Digital images of the tibial growth plate morphology were captured with the Leica DM1000 LED (Leica, Germany).

### Co-IP assay

The detailed Co-IP protocol is outlined in the instructions of the previous study [66]. In brief, after 48 h of transfection with the pCMV-N-Flag or pCMV-Cant1-Flag vector, cell proteins were extracted using cell lysis buffer (P0013, Beyotime, China) and incubated with anti-Flag magnetic beads (no. P2115, Beyotime, China) for 4 h at room temperature. Following incubation, the bound proteins were denatured for Western blotting.

### Statistical procedures and data analysis

Data visualization was performed using GraphPad Prism 9.5 (GraphPad Inc., USA). Statistical differences were evaluated with an unpaired 2-tailed Student’s *t* test and one-way analysis of variance (ANOVA). A *P* value less than 0.05 was considered statistically significant. Data are presented as mean ± SD.

## Data Availability

The data that support the findings of this study are available from the corresponding authors upon reasonable request.
