# Erratum to “Dysregulation of the Cant1/beta-Catenin/TCF4-CHSY1 Axis Underpins Impaired ECM Biosynthesis in Skeletal Disorders”

**DOI:** 10.34133/research.1354

**Published:** 2026-07-08

**Authors:** Yuanliang Li, Wenqi Yu, Yingxin Li, Kai Liu, Wenjing Xu, Cong Li, Yugu Li, Ying Li, Zhaoxin Tang, Yung-Fu Chang, Aoyun Li, Hui Zhang

**Affiliations:** ^1^ College of Veterinary Medicine, South China Agricultural University, Guangzhou 510642, China.; ^2^ College of Veterinary Medicine, Cornell University, Ithaca, NY, USA.; ^3^ College of Veterinary Medicine, Henan Agricultural University, Zhengzhou 450046, China.; ^4^ College of Animal Science, Xizang Agricultural and Animal Husbandry University, Linzhi 860000, China.

In the Research Article “Dysregulation of the Cant1/β-Catenin/TCF4-CHSY1 Axis Underpins Impaired ECM Biosynthesis in Skeletal Disorders” [1], errors occurred in Figures 2 and 4.

**Fig. 2.**
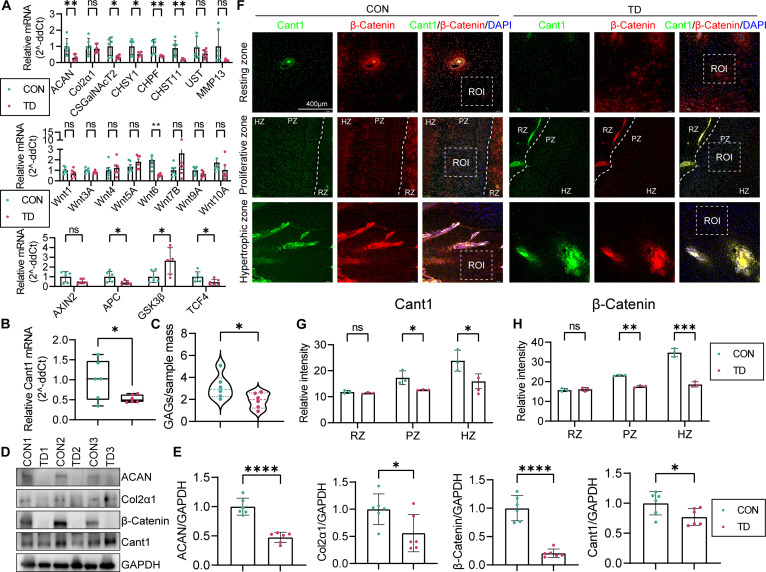
Mechanisms of ECM biosynthesis disorders and abnormal Wnt/β-Catenin signaling pathway transduction in TD models. (A) mRNA levels of ECM biosynthesis factors and canonical Wnt/β-Catenin signaling pathway components; (B) mRNA levels of Cant1; (C) Quantitative analysis of GAG content; (D) Protein blots; (E) Protein levels of ACAN, Col2α1, β-Catenin, and Cant1; (F) Digital immunofluorescence images of CON tibia and TD tibia; (G-H) Relative fluorescence intensity of Cant1 and β-Catenin in ROI of CON tibia and TD tibia.

**Fig. 4.**
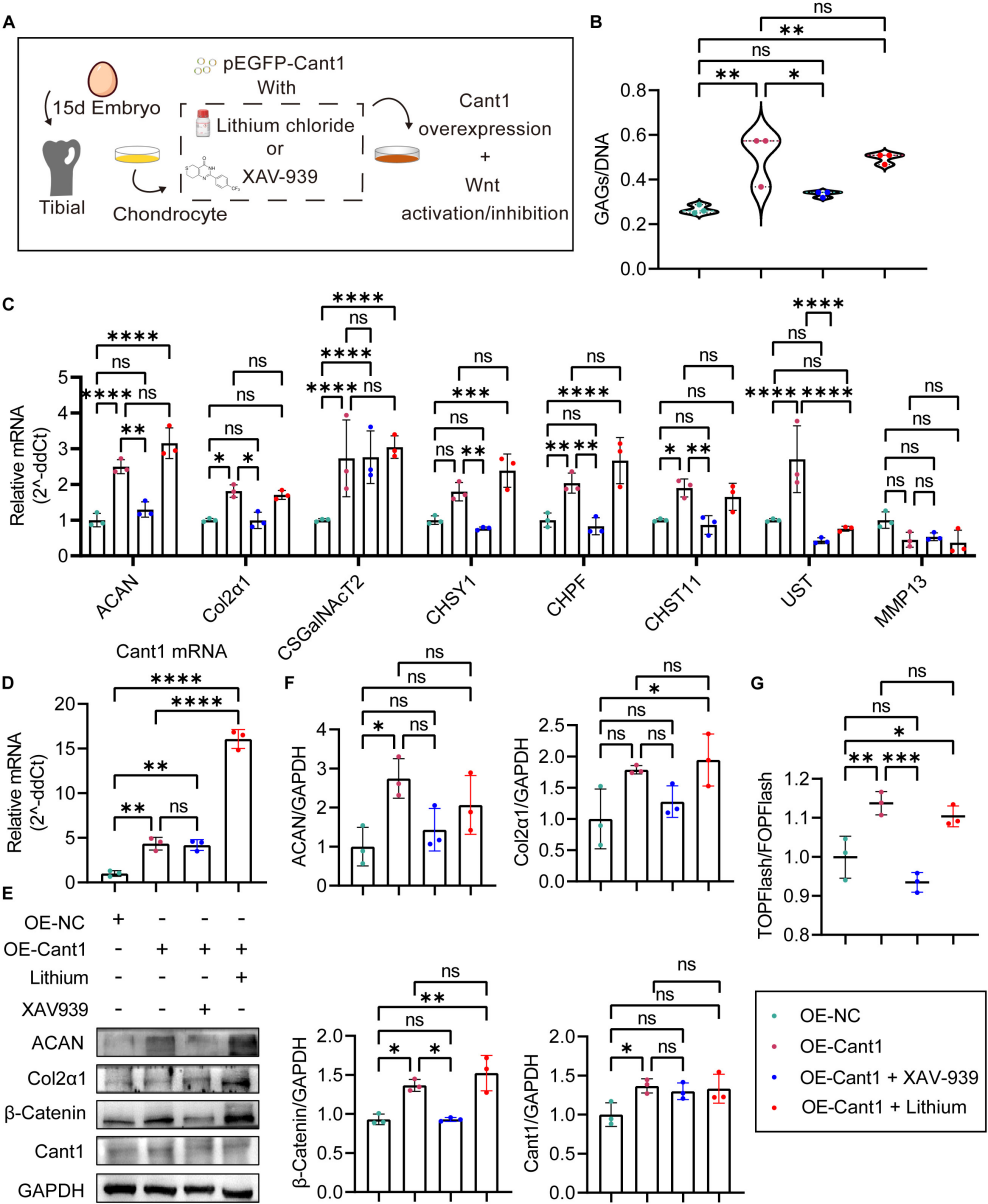
The inhibition of the canonical Wnt/β-Catenin signaling pathway downregulates ECM biosynthesis promoted by Cant1 overexpression. (A) Illustration of the cell model for Cant1 overexpression combined with activation or inhibition of the canonical Wnt/β-Catenin signaling pathway; (B) Quantitative analysis of GAG content; (C) mRNA levels of ECM biosynthesis factors; (D) mRNA levels of Cant1; (E) Protein blots; (F) Protein levels of ACAN, Col2α1, Cant1, and β-Catenin; (G) Wnt/β-Catenin transcriptional activity (TOP/FOPFLASH ratio)

During the final submission, the authors inadvertently uploaded an older version of Figure 2. The corrected version of Figure 2, with a bar chart showing the mRNA expression levels of Wnt ligands in Figure 2A, replaces the old version of Figure 2A, which did not include this bar chart.

During the final submission, the authors inadvertently uploaded an older version of Figure 4. Figure 4A has been corrected with a 15-day embryo. In Figure 4E, the grouping label (+/- label) was incorrect.

The figures have now been corrected in the original HTML and PDF versions of the article.
