# Supplementary material for: Dysregulation of the Cant1/β-Catenin/TCF4–CHSY1 Axis Underpins Impaired ECM Biosynthesis in Skeletal Disorders
Source: Research (Wash D C). 2026 Apr 1;9:1227. doi: 10.34133/research.1227 (PMC13039522; doi:10.34133/research.1227)
Supplement: Supplementary 1 — Figs. S1 to S3 Tables S1 to S5 [file research.1227.f1.docx]

**Table S1**. Criteria for gait score assessment for broilers.

| Gait score | Criteria |
| --- | --- |
| 0 | Normal, smooth, and agile. The toes are furled while raised |
| 1 | Slightly abnormal, but unidentifiable |
| 2 | Definite and identifiable abnormality, but has limited effects on walking ability |
| 3 | Obvious abnormality affects the ability to move. The bird has imbalanced steps and squats within 15 s |
| 4 | Severe abnormality, but still capable of walking. The bird takes more than 5 s to rise when nudged, and squats after a few steps |
| 5 | Incapable of walking |

**Table S2**. List of used primers for RT-qPCR

| Name | Primer 5’ → 3’ |
| --- | --- |
| GAPDH | F: AGTCAACGGATTTGGCCGTA |
|  | R: TTCCCGTTCTCAGCCTTGAC |
| ACAN | F: CACCTGGTGTGAGGACCATC |
|  | R: AGCTCTGAAGCAGAAAGCGT |
| AXIN2 | F: CAAGCGACGAGTTTGACTGC |
|  | R: CCTTCCTTCGTACATGGGCA |
| APC | F: TGGCCGCAGCTTCTTATGAT |
|  | R: AGCTGTTTCAGCACCTCCTT |
| Cant1 | F: AAGGTTGTCGGCTACAAGGG |
|  | R: TCAAAAGATGCTGGGGGTCG |
| CHPF | F: TGCCGGATGAACACCATCAA |
|  | R: TGGAAGACATCGCGGTCAAA |
| CSGalNAcT2 | F: AAGAGCCCCAAGGTCAATGG |
|  | R: GACGCCGTATTCACTAGGCA |
| CHSY1 | F: TCTTTCTTGGACAGACCGGC |
|  | R: CTCCCGGCTCATGATCACTC |
| CHST11 | F: ATCCTCGTCATCTTCTATTTC |
|  | R: ATACAGTTCCTGGAGTGGG |
| Col2α1 | F: GCCACCCTCAAATCCCTCAA |
|  | R: ACTCGGGATGGCAGAGTTTG |
| GSK3β | F: ACTGCAGTCCTATGGAGTTGA |
|  | R: ATATGCCACATCCTCTGCACAT |
| MMP13 | F: TTTGGGCTATGAATGGCTAT |
|  | R: TAGTATGCAGGATGCGGACA |
| UST | F: TCGTATCAGGAAGCCACGTC |
|  | R: GCACCACTGGCTGTATCCAT |
| Wnt1 | F: TGCAGACGGGGATTTGCTAC |
|  | R: TGATGGTCTGCATCAGCTCC |
| Wnt3A | F: AAGAGCCCCAAGGTCAATGG |
|  | R: GACGCCGTATTCACTAGGCA |
| Wnt4 | F: TGTGACCACGACCTCAAGAA |
|  | R: ACCAGTGGAATTTGAAGCTG |
| Wnt5A | F: AGGTGCTCTGGGGACACTT |
|  | R: TGGGGTTCATAGGGTTCATC |
| Wnt6 | F: GACGTGCAGTTTGGCTATGA |
|  | R: GCATTTGCACTCTGTCCTCA |
| Wnt7B | F: TGTTTTACCGTTTGGAGCGG |
|  | R: TCCCAGTCAAGAGCGTTTCG |
| Wnt9A | F: GGCCTTCCTCTACGCCATTT |
|  | R: GCTTCCTCCCCAAGAACTCC |
| Wnt10A | F: CACAACATCCTGCGGCAAAC |
|  | R: CTGACCCACTCGGTGATGC |
| TCF4 | F: AGGGATAAGCAGCCAGGAGA |
|  | R: TCTTAGGAGCGCTCAGGTCT |

**Table S3**. RNA interference sequence

| Name | Primer 5’ → 3’ |
| --- | --- |
| siCant1 | Sense: GGUUGUCUUCAAUGGGAAGTT |
|  | Antisense: CUUCCCAUUGAAGACAACCTT |
| siCHSY1 | Sense: GGCAAGGUUGAAUUCUUCUTT |
|  | Antisense: AGAAGAAUUCAACCUUGCCTT |
| siNC | Sense: UUCUCCGAACGUGUAACGUTT |
|  | Antisense: ACGUGACACGUUCGGAGAATT |

**Table S4**. Primers for DNA sequence

| Name | Primer 5’ → 3’ |
| --- | --- |
| EGFP-Cant1 cds | F: TCCGCTAGCGCTACCGGACTCAGATCTATGATGTGCAGGCTCTCCTCAC |
|  | R: GCGGTACCGTCGACTGCAGAATTCTCAGGTAACCTGCAGCAGGGA |
| Flag-Cant1 cds | F: AGCCCGGGCGGATCCAAGCTTATGATGTGCAGGCTCTCCTCAC |
|  | R: GATCTGTCGACGATATCGAATTCTCAGGTAACCTGCAGCAGGGA |
| Flag-TCF4 cds | F: AGAGCCCGGGCGGATCCATGCCGCAGCTGAACGGC |
|  | R: CTAGTTCTAGACTCGAGCTATTCTAAGGACTTGGTTA |
| pGL6-CHSY1 promoter 2000bp | F: TGGCCTAACTGGCCGGTACCATCGGAGCTCCTGTTGTTGTCT |
|  | R: TCTAAGCTTCTGCAGATCTGCTGCTGGGAGAGCTGAT |
| pGL6-CHSY1 promoter 1500bp | F: GCCTAACTGGCCGGTACCCCCACATCTTAATTGAGTGCATCGTT |
|  | R: TCTAAGCTTCTGCAGATCTGCTGCTGGGAGAGCTGAT |
| pGL6-CHSY1 promoter 1000bp | F: GCCTAACTGGCCGGTACCATAAAACCCTCGGCTTTAATTGCCCT |
|  | R: TCTAAGCTTCTGCAGATCTGCTGCTGGGAGAGCTGAT |
| pGL6-CHSY1 promoter 500bp | F: CCTAACTGGCCGGTACCCCCCATCACCAGTCCACCA |
|  | R: TCTAAGCTTCTGCAGATCTGCTGCTGGGAGAGCTGAT |

**Table S5**. Primers for site mutation

| Name | Primer 5’ → 3’ |
| --- | --- |
| CHSY1-MUT1 | F: TTGGAGAggggAGACAAAAGGAAAAAATCAAAGCC |
|  | R: TTTGTCTccccTCTCCAAGGAGACAGAGAAGCAG |
| CHSY1-MUT2 | F: GAACAAAGAggggAGGAAAAAATCAAAGCCACACC |
|  | R: TTCCTccccTCTTTGTTCTCCAAGGAGACAGAGA |
| CHSY1-MUT3 | F: GAAAAAAggggAGCCACACCTCTGTGCTTTGTT |
|  | R: TGTGGCTccccTTTTTTCCTTTTGTCTTTGTTCTCC |


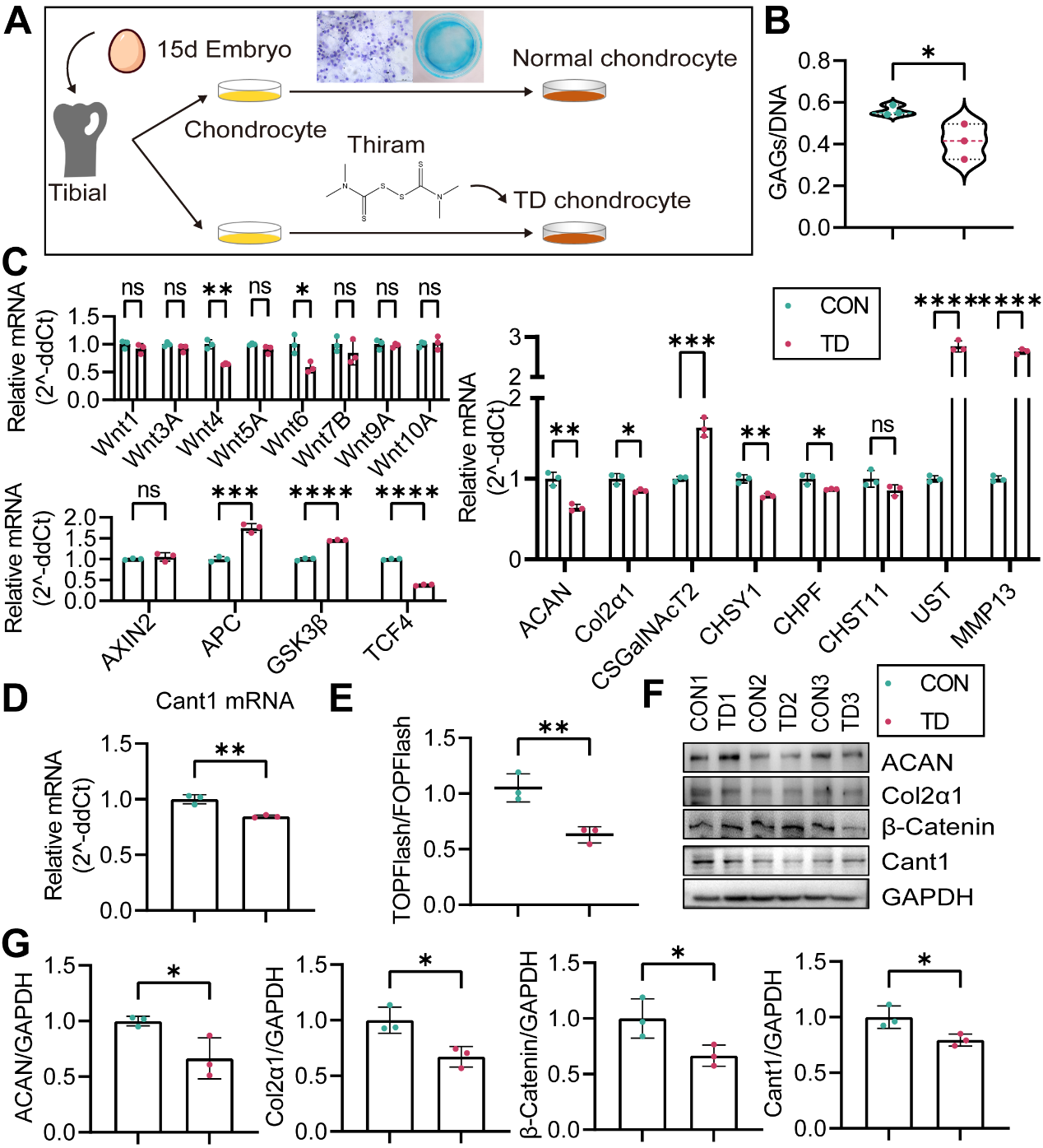


**Figure S1. Mechanism of ECM biosynthesis disorders and abnormal canonical Wnt/β-Catenin signaling pathway transduction in TD chondrocytes.** (A) The illustration of TD model establishment; (B) Quantitative analysis of GAGs content; (C) The mRNA levels of ECM biosynthesis factors and canonical Wnt/β-Catenin signaling pathway; (D) The mRNA levels of Cant1; (E) Canonical Wnt/β-Catenin signaling pathway transcriptional activity (TOP/FOPFLASH ratio); (F)The protein blots; (G) The protein levels of ACAN, Col2α1, β-Catenin, and Cant1. ns *p* > 0.05, ⁎*p* < 0.05, ⁎⁎*p* < 0.01, ⁎⁎⁎*p* < 0.001 vs CON group, the same below.


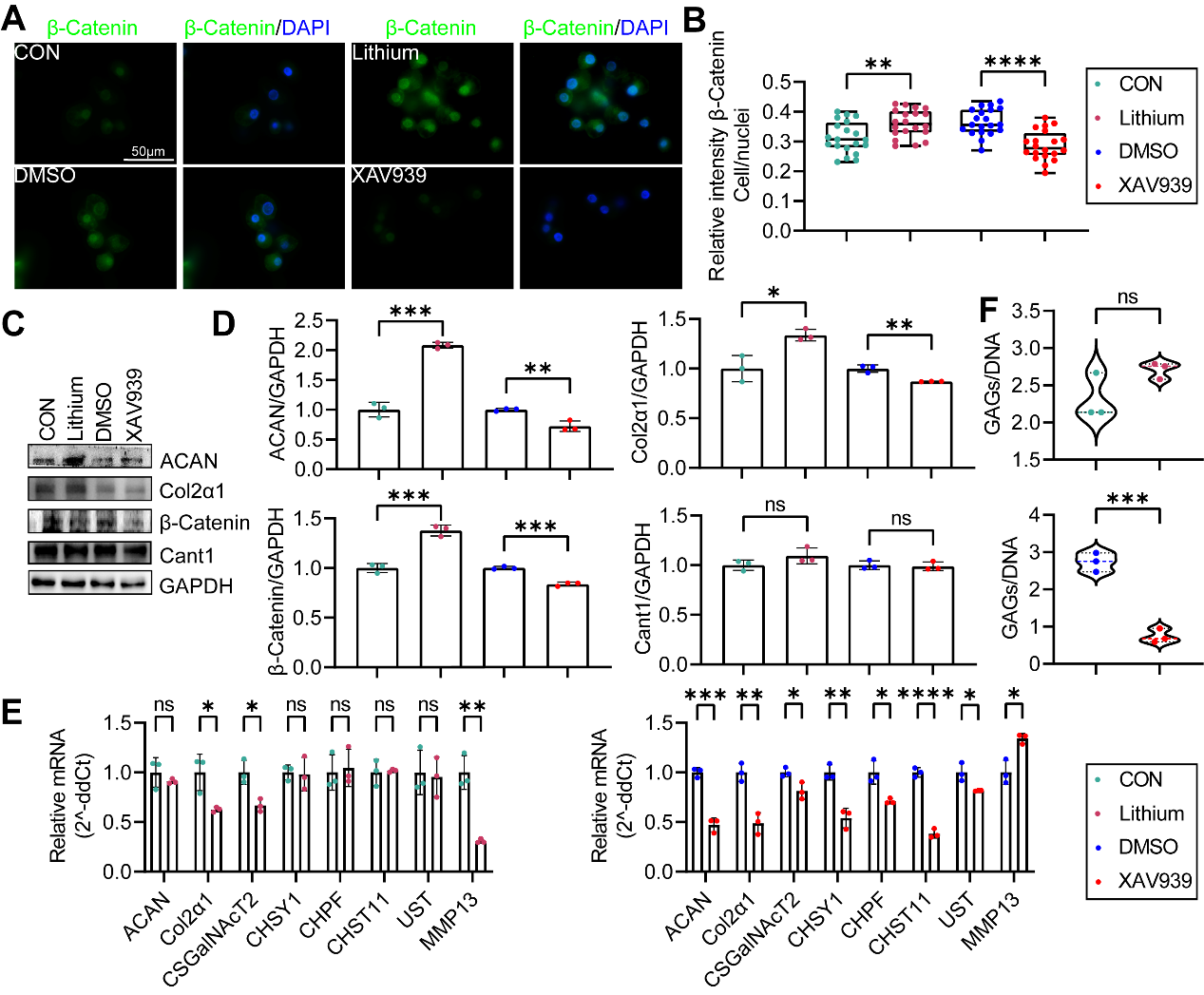


**Figure S2. Regulation of canonical Wnt/β-Catenin signaling pathway on ECM biosynthesis in chondrocytes.** (A-B) The β-Catenin relative indensity in broiler chondrocytes; (C) The protein blots; (D) The protein levels of ACAN, Col2α1, Cant1, and β-Catenin; (E) The mRNA levels of ECM biosynthesis factors; (F) Quantitative analysis of GAGs content.


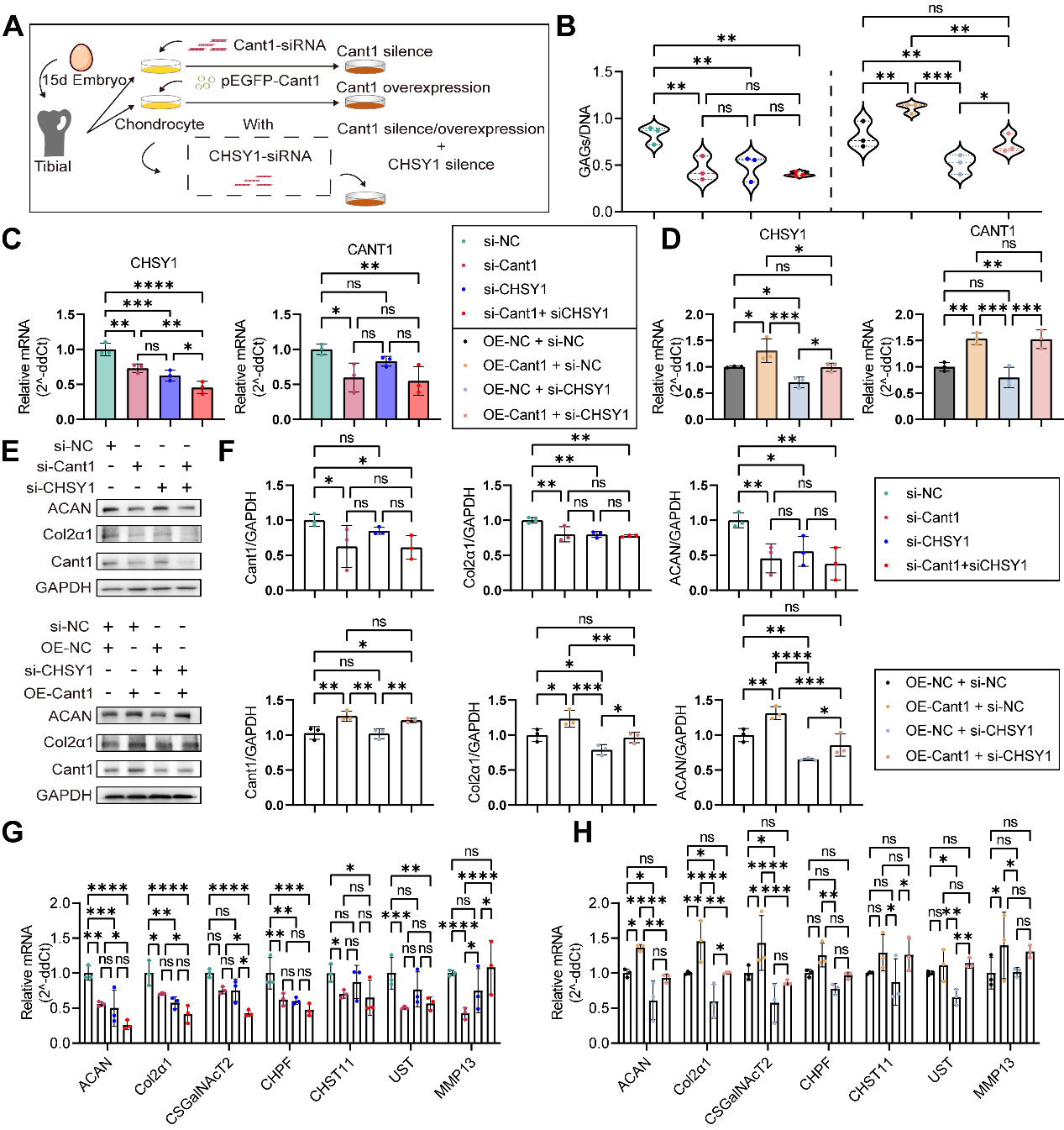


**Figure S3. CHSY1 act as a downstream factor synergistically regulates ECM biosynthesis in chondrocytes with Cant1.** (A) The illustration of cell model construction of Cant1 silence/overexpression combined with CHSY1 silence; (B) Quantitative analysis of GAGs content; (C-D) The mRNA levels of CHSY1 and Cant1; (E) The protein blots; (F) The protein levels of ACAN, Col2α1, and Cant1; (G-H) The mRNA levels of ECM biosynthesis factors.
